# Resolution-Based Therapies: The Potential of Lipoxins to Treat Human Diseases

**DOI:** 10.3389/fimmu.2021.658840

**Published:** 2021-04-23

**Authors:** Rafael I. Jaén, Sergio Sánchez-García, María Fernández-Velasco, Lisardo Boscá, Patricia Prieto

**Affiliations:** ^1^ Instituto de Investigaciones Biomédicas Alberto Sols, CSIC-UAM, Madrid, Spain; ^2^ Centro de Investigación Biomédica en Red de Enfermedades Cardiovasculares (CIBER-CV), Instituto de Salud Carlos III, Madrid, Spain; ^3^ Instituto de investigación del Hospital la Paz, IdiPaz, Madrid, Spain; ^4^ Departamento de Farmacología, Farmacognosia y Botánica, Facultad de Farmacia, Universidad Complutense de Madrid, Madrid, Spain

**Keywords:** lipoxin, inflammation, oxidative stress, lipid mediators, pathology

## Abstract

Inflammation is an a physiological response instead an essential response of the organism to injury and its adequate resolution is essential to restore homeostasis. However, defective resolution can be the precursor of severe forms of chronic inflammation and fibrosis. Nowadays, it is known that an excessive inflammatory response underlies the most prevalent human pathologies worldwide. Therefore, great biomedical research efforts have been driven toward discovering new strategies to promote the resolution of inflammation with fewer side-effects and more specificity than the available anti-inflammatory treatments. In this line, the use of endogenous specialized pro-resolving mediators (SPMs) has gained a prominent interest. Among the different SPMs described, lipoxins stand out as one of the most studied and their deficiency has been widely associated with a wide range of pathologies. In this review, we examined the current knowledge on the therapeutic potential of lipoxins to treat diseases characterized by a severe inflammatory background affecting main physiological systems, paying special attention to the signaling pathways involved. Altogether, we provide an updated overview of the evidence suggesting that increasing endogenously generated lipoxins may emerge as a new therapeutic approach to prevent and treat many of the most prevalent diseases underpinned by an increased inflammatory response.

## Introduction

**Figure 1:**
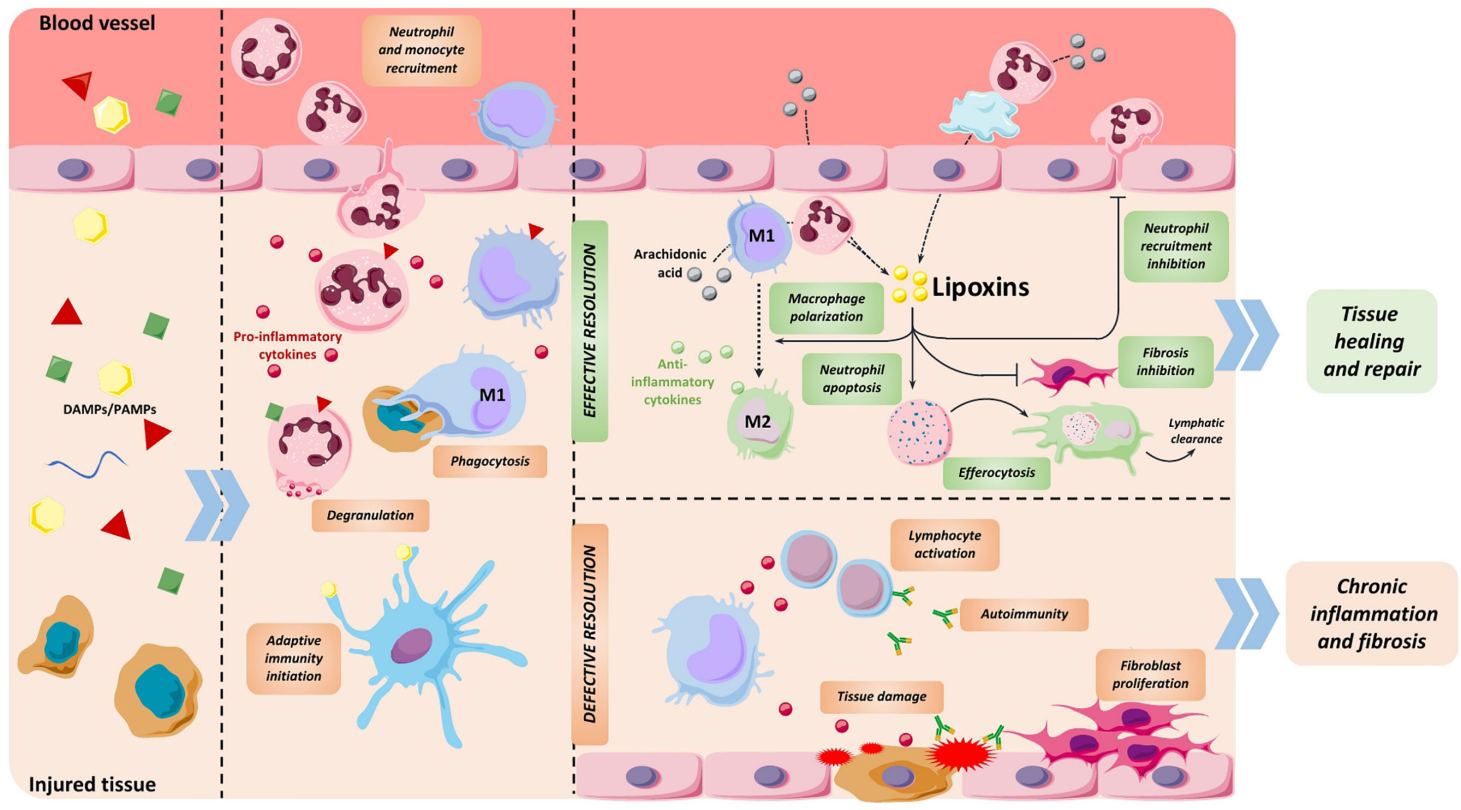
Stages of inflammatory process. Release of pathogen and damage-associated molecular patterns (PAMPs and DAMPs) from injured tissue initiates inflammation by promoting the recruitment of neutrophils followed by monocytes. These immune cells blunt the source of damage by exerting different functions like degranulation to release toxic substances, phagocytosis, release of pro-inflammatory cytokines and initiating antigen recognition. At the end of this phase, immune cells shift to a resolutive phenotype, generating LXs and other specialized pro-resolving mediators that support the end of the inflammatory response. During resolution, these compounds prevent neutrophil recruitment and induce their apoptosis, polarize macrophages towards an anti-inflammatory phenotype, promote efferocytosis and inhibit fibroblast proliferation, among other functions. As a result, tissue is appropriately repaired. If the resolution phase is defective or insufficient, pro-inflammatory stimuli persist and end up damaging the tissue, causing chronic inflammation and subsequent fibrosis and organ dysfunction.

Inflammation can be defined as the physiological response initiated by cells and tissues that aims to protect the organism against infections or injuries caused by exogenous or endogenous agents (1). Interestingly, it also plays an important role in processes like ovulation (2) or physiological interactions with microbiota (3). Acute inflammation entails two stages: an initial phase that comprises the onset of the inflammatory reaction to eliminate the danger signal, and a subsequent resolution phase wherein inflammation is blunted to restore homeostasis (**Figure 1**). Different immune cell populations (i.e., macrophages, neutrophils, lymphocytes) and related mediators (cytokines, eicosanoids, immunoglobulins, among other) establish an orchestrated system that mediates these two phases as well as the transition between them (1, 4).

Within the molecules that actively participate in the resolution phase, special attention has been paid to specialized pro-resolving mediators (SPMs) (4), which promote resolution of inflammation by reducing the levels of pro-inflammatory cytokines and reactive oxygen species (ROS) and by modulating correct tissue repair (5–9), among other functions. So far, four types of SPMs have been classified according to the precursor molecule they originate from and the enzyme implicated in their metabolism (**Table 1**). These include lipoxins (LXs), which derivate from omega 6 arachidonic acid and are the subject of this review, and other SPMs such as resolvins, maresins and protectins, that are structurally distinct and result from a different biosynthetic pathway derived from omega 3 fatty acids. Despite having similar pro-resolving actions, they can exert their function through different receptors driving to alternative signaling pathways [for review (10)].

**Table 1:** Classification of principal specialized pro-resolving lipid mediators.

SPM	Main enzymes	Main precursor molecule	References
**Lipoxins**	5-, 12- and 15-lipoxygenases	Arachidonic Acid (AA)	(1)
**Aspirin-Triggered Lipoxins**	Acetylated COX-2 Cytochrome P450 enzymes		
**D-Series Resolvins**	Acetylated COX-2 5- and 15-lipooxygenases	Docosahexaenoic acid (DHA)	(2)
**E-Series Resolvins**	Acetylated COX-2 Cytochrome P450 enzymes 5- and 15-lipoxygenases	Eicosapentaenoic Acid (EPA)	(2, 3)
**Protectins**	Acetylated COX-2 15-lipooxygenase	Docosahexaenoic acid (DHA)	(4)
**Maresins**	12-lipoxygenase		(5)

**Figure 2:**
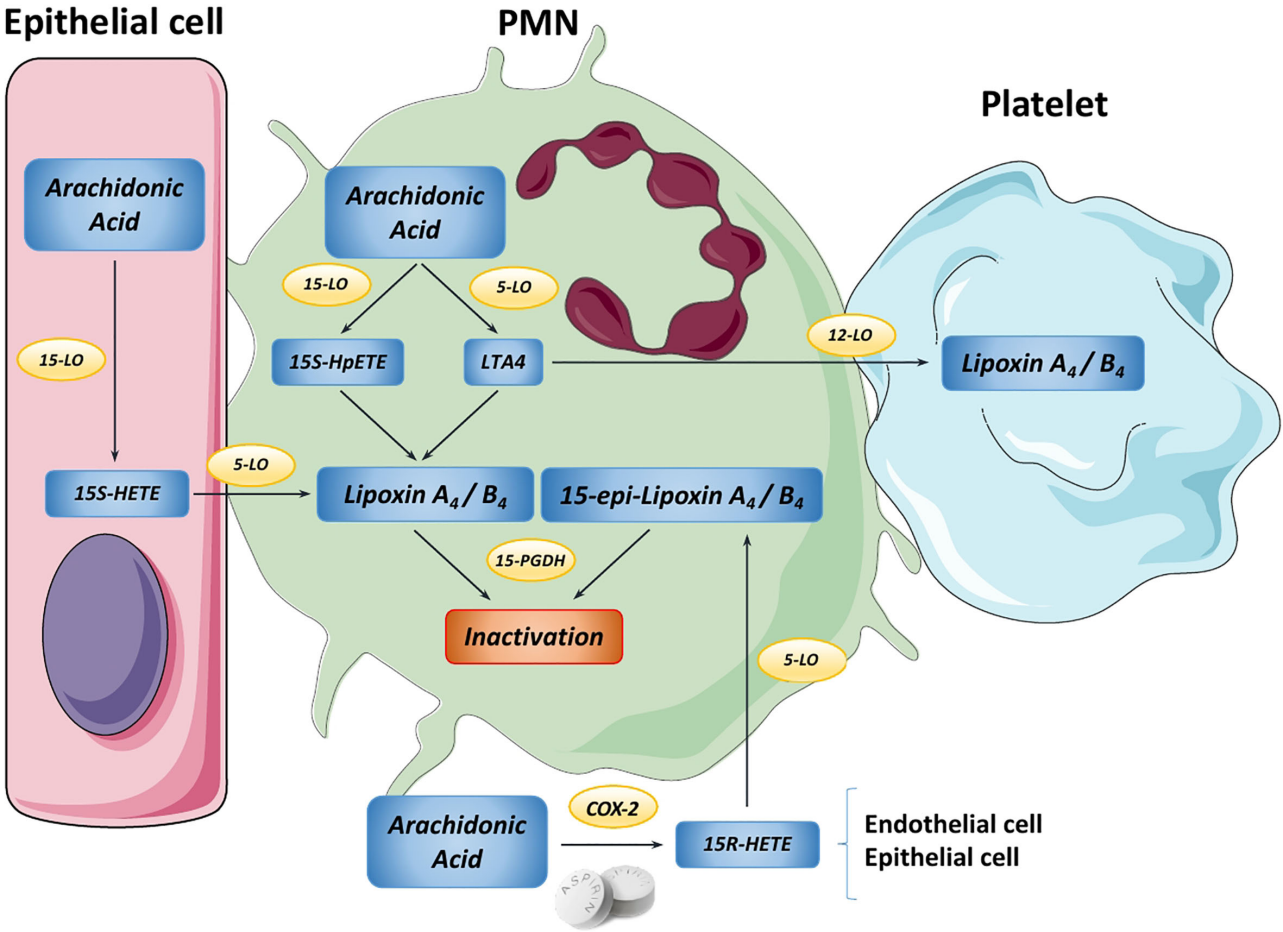
Overview of the main lipoxin biosynthetic pathways. In the PMN cell, Arachidonic Acid (AA) can be converted into either 15S-HpETE or Leukotriene A_4_ (LTA4) by 15-LO and 5-LO, respectively. Then, these metabolites are transformed into native lipoxin A_4_ or B_4_. Moreover, platelets can use LTA_4_ from PMNs to produce LXA_4_/B_4_ in a process known as transcellular biosynthesis. Likewise, in epithelial cells AA is transformed by 15-LO into 15-HETE, which is transferred to PMNs to produce LXA_4_/B_4_. Endothelial cells can use AA to yield the intermediate 15R-HETE by COX-2, step that is promoted by aspirin. This transitional molecule is metabolized by PMNs to generate the “aspirin-triggered” 15-epi-lipoxins A_4_ and B_4_. Finally, both native and aspirin-triggered LXs can be rapidly inactivated by 15-PGDH, stopping the downstream signaling.

LXs were the first SPM to be described (11) and the most studied to date since their role has been found to be essential in various inflammatory diseases (5, 12). Generation of native LXA_4_ and LXB_4_ results from the sequential lipoxygenation of the arachidonic acid present in the lipid membrane by the action of 5, 12 and/or 15-lipoxygenases (13) (**Figure 2**). An alternative biosynthetic route requiring aspirin-mediated acetylation or statin-induced nitrosylation of COX-2 generates LX 15-R-epimers called 15-epi-lipoxins or aspirin-triggered lipoxins (ATLs) (14, 15). Both pathways of LX biosynthesis are driven by the coordinate interaction of distinct cell types such as neutrophils, eosinophils, macrophages, endothelial cells, epithelial cells, parenchymal cells or platelets in a process known as transcellular biosynthesis, which also occurs in the synthesis of pro-inflammatory eicosanoids (16). Transcellular biosynthesis allows cells to rapidly switch the production of pro-inflammatory mediators to anti-inflammatory based on the distinct cell types present in their environment, thus adapting eicosanoid synthesis to inflammatory or resolutive contexts (17).

LXs mainly exert their functions by binding with high affinity to a G-protein coupled receptor named N-formyl peptide receptor 2 (FPR2), also called formyl peptide receptor-like 1 (FPRL1) or ALX receptor (ALXR) (18). ALXR is expressed in a wide array of tissues, including bone marrow, brain, lung, gastrointestinal tract and heart (18) and activates a plethora of cell type-specific pathways (12). Consequently, LXs are able to modulate a large variety of processes showing diverse actions depending on the cell type they act on (8). Among their functions, LXs are capable of blocking the arrival of excessive PMNs to the inflammatory site (19–22) and switching macrophage phenotype from pro-inflammatory (M1) to anti-inflammatory (M2), stimulating efferocytosis and repair-associated mechanisms (23–25). SPMs can also blunt the cytotoxicity of NK cells (26) and decrease antibody production and proliferation in memory B cells preventing maladaptive immunity and autoimmune reactions (27). They also affect non-immune cells; for example, they elicit anti-fibrotic responses by repressing metalloproteinases (MMP) and inducing tissue inhibitors of metalloproteinase (TIMPs) expression in fibroblasts (28). In cardiomyocytes, they have been described to induce the antioxidant NRF2 pathway, thus reducing damage from hypoxia/reoxygenation (29). The recently developed Atlas of Inflammation Resolution (AIR) is a web resource that gathers updated data on the various processes modulated by LXs and other SPMs with in-depth information about the underlying molecular pathways and their interactions (30).

The effect of LXs is destined to be local and transitory, therefore they are rapidly metabolized and inactivated by modifications at different carbons by distinct enzymes, primarily 15-hydroxyprostaglandin dehydrogenase (PGDH) (12, 31). Since this inactivation is stereospecific, ATL degradation occurs at approximately 50% of the conversion rate of native LXA_4_, resulting in an increased half-life (32). However, to further prolong LX action, synthetic LX analogs, such as LXA_4_-methyl ester (LXA_4_-ME), benzo-LXA_4_ and 15-epi-16-(p-fluoro)-phenoxy-​lipoxin A_4_ (ATLa), have been designed by substitutions at different carbons (33). These analogs resisted rapid conversion while retaining their intrinsic properties and biological functions (31), resulting in a more potent bioactivity (18).

It is now widely accepted that a defective resolution phase may lead to chronic inflammation, a more persistent response that eventually causes tissue fibrosis, necrosis and irreversible damage, severely affecting appropriate functioning of organs (4). Impaired resolution may be enhanced by reduced dietary intake of fatty acids, by genetic polymorphisms affecting SPM biosynthesis and SPM receptors functionality and expression, and by abnormal downstream signaling upon receptor activation (34). In recent years, chronic inflammation has been demonstrated to underpin pathologies not previously thought to be inflammatory, like atherosclerosis, Alzheimer’s disease, cardiovascular diseases or even cancer (35, 36), which underscores the importance of an effective and tightly regulated resolution process. Indeed, current therapies for these diseases include treatment with generic corticosteroids and non-steroidal anti-inflammatory drugs (NSAIDs), however their main drawbacks are their potential side-effects, including hyperglycemia, hypertension, osteoporosis, increased bleeding or even neurological alterations and treatment resistance in certain patients (37, 38). As an alternative, LXs and other SPMs arise as an effective anti-inflammatory treatment with reduced side effects (39). Furthermore, reduced circulating levels of LXs which reach nanomolar concentrations under physiological conditions- have been related to worsened prognosis and disease progression, suggesting that LXs can serve as predictive biomarkers as observed in sputum from severe asthma patients (40), urine from lupus patients (41) or plasma from tuberculosis patients (42). In the following sections, we will provide an up-to-date analysis in relation to the current research on the role of LXs in pathologies affecting the main physiological organ systems.

## Neurological Diseases

### Alzheimer’s Disease

Alzheimer’s disease (AD) is a neurodegenerative disorder representing the most common cause of dementia in the elderly. It is characterized by the loss of cognitive functioning (thinking, remembering, and reasoning) and behavioral skills to such an extent that it interferes with a person’s life and daily activities. Although its causes are not fully understood, the deposition of toxic β-amyloid and P-Tau aggregates in the brain has been reported to interfere with neuronal circuits and activate pro-inflammatory signaling in microglia, thus initiating brain damage (43, 44).

Since both neurons and glia express ALXR (45), increasing LXs production in the brain may blunt inflammation and ameliorate the outcome of AD patients. Interestingly, LXA_4_ levels in brains from AD patients are reduced in contrast to healthy controls (n=10), suggesting that AD-associated neuroinflammation may be worsened by defective resolution mechanisms (45). In fact, treatment with LXs and their derivatives exerted beneficial effects in a human microglial cell line (46) and protected against the harmful accumulation of both β-amyloid and P-Tau aggregates by improving their phagocytic elimination *via* upregulation of IL-10 and TGF-β anti-inflammatory pathways in different mouse models of AD by either s.c. or intracerebroventricular (ICV) injection (47–50). These beneficial effect observed upon LX treatment can be partially explained by the decreased activity of the NF-κB/IL-1β pathway, which in turn downregulates p38, ERK, JNK and GSK3β kinases responsible for neuroinflammation and Tau phosphorylation (49, 51–53).

### Stroke

Stroke is one of the leading causes of death worldwide and of disability in adults. Ischemic stroke is the most prevalent and occurs when a brain artery is totally or partially blocked by a clot or burst, prompting ischemia and preventing the brain from receiving oxygen and nutrients and triggering an inflammatory response that causes severe brain damage (54). Within the most common symptoms, patients exhibit trouble with speaking and understanding, paralysis in the face, arms or legs, partial blindness, loss of coordination and headache (54). Current therapies are limited, have side effects and their efficiency depends on a narrow time window, therefore research on alternative treatments is mandatory (54, 55). In addition, within the first five years after stroke, a high percentage of patients develop depressive symptoms, worsening their prognosis (56). Interestingly, it has been shown that circulating LX levels are lower in patients with ischemic stroke (n= 75 patients *vs*. 35 healthy) (57) and with depression compared to controls (n=143 patients *vs*. 44 healthy) (58). Concordantly, LXs and their analogs have been extensively demonstrated to play an important protective role in animal models of stroke (55). Thus, ICV inoculation of LXA_4_-ME immediately after occlusion reduced neurological dysfunction, infarct volume and histological damage in a rat model (59, 60). LXA_4_-ME treatment managed to decrease the number of apoptotic neurons (59) and to inhibit neutrophil infiltration and microglia activation, overall reducing pro-inflammatory cytokine levels mainly by downregulating NF-κB pathway (60). Both LXA_4_-ME (ICV) and BML-111 –ALXR analog- (i.v.) treatment allowed for the maintenance of blood-brain barrier, since MMP-9 and MMP-3 expression and activity were decreased whereas TIMP-1 was increased, further protecting against cerebral ischemia (55, 61, 62). Similar results were observed with ATL (i.v.), which also prevented leukocyte-platelet aggregations within cerebral microvasculature, therefore reducing the risk of cerebral atherothrombosis (63). Interestingly, treatment with rosiglitazone, a PPARγ agonist, promoted neuroprotection in a murine model of stroke in part by inducing 5-LO synthesis, thus increasing LXA_4_ (64). Other molecular pathways implicated in LX-associated neuroprotection are NRF2/HO-1 and autophagy, both involved in the antioxidant response (65, 66).

Generally, all these data support the idea that LX treatment emerge as a new therapeutic tool for brain diseases since these compounds can control increased inflammation and oxidative stress associated with brain damage. More clinical and experimental approaches are needed to elucidate the potential and the specific mechanism behind LX-mediated neuroprotection. Thus, additional data on the anti-apoptotic and pro-survival actions of LX in neurons or their role in the maintenance of the blood-brain barrier can reveal the mechanisms supporting LX-dependent neuroprotection.

## Cardiovascular Diseases

### Atherosclerosis

Atherosclerosis, the formation of fibro-fatty lesions in the artery wall, causes much morbidity and mortality worldwide as it is commonly associated with myocardial infarction or stroke, as well as peripheral artery disease. The main risk factors include hypercholesterolemia and blood lipid dysregulation but also hypertension, cigarette smoking and diabetes mellitus. Increasing evidence points to a role for the immune system, being increased pro-inflammatory mediators emerging factors for the prognosis of patients (36, 67).

Current research has revealed that deficient LX levels are tightly related to atheroma development. In fact, LX precursors profile was found reduced in unstable atherosclerotic plaques compared to stable plaques, suggesting that changes on pro-resolving lipids may promote plaque inflammation and rupture (68). Studies in rabbit atherosclerotic arteries showed reduced levels of LXs along with exacerbated levels of pro-inflammatory cytokines associated to a defective resolution process during atherogenesis (69, 70). Interestingly, statins, the most prevalent atherosclerosis medication, can upregulate the lipoxygenase pathway (71), and simvastatin, atorvastatin and lovastatin treatments were found to promote 15-epi-LXA_4_ formation independently of aspirin, indicating that synthesis of 15-epi-LXA_4_ could also be statin-triggered (15).

Although the molecular mechanism of LX-associated atheroprotection is not completely known, evidence suggests that LXs modulate the inflammatory process within atherosclerotic plaques (foam cells, ROS, cytokines) as well as elements interacting with them, such as vascular smooth muscle and immune cells, resulting in reduced necrosis and increased plaque stability (72). LXs treatment has been demonstrated to inhibit foam cell formation and apoptosis in macrophages mainly by blunting both the expression and signaling of CD36, the main receptor involved in oxLDL uptake (69). In addition, i.p. administration of LXA_4_ and benzo-LXA_4_ reduced aortic expression of pro-inflammatory cytokines (IL-1β and IL-6) and adhesion molecules (MCP-1, VCAM-1 and ICAM-1) in a mouse model of atherosclerosis (73). ATL was also found to inhibit vascular smooth muscle cell migration, preventing atherosclerotic lesions (74).

Therefore, the atheroprotective mechanisms elicited by LXs include reduced infiltration of inflammatory cells that can modulate the promotion of pro-resolution phenotypes inside the plaque. The evidence of statin-triggered LXs may explain some of the anti-inflammatory effects of statin therapy (75) and represents an interesting approach to increase LX levels and promote resolution in atherosclerotic patients especially by inducing plaque stabilization and preventing the appearance of acute cardiovascular events. However, additional studies are required to completely understand their synthesis mechanism as well as the resolution potential of statin treatment. Lastly, LXs role in plaque stabilization by preventing rupture and adverse thrombotic events is of paramount relevance in the context of atherosclerosis and it will undoubtedly be addressed in future research.

### Myocardial Ischemia/Reperfusion Injury

Myocardial ischemia/reperfusion (I/R) injury occurs due to blood restoration after a critical period of coronary artery obstruction, which is associated with clinical interventions such as thrombolysis, angioplasty, and coronary bypass surgery. This reperfusion injury involves the activation of an inflammatory cascade and is manifested as functional impairment, arrhythmia, and accelerated progression of cell death in certain critically injured myocytes. Among the main mediators of reperfusion injury are oxygen radicals, calcium mishandling, and excessive inflammation (76). The primary therapeutic approach consists of improving the blood flow to the cardiac muscle and establishing a medical treatment for the main symptoms caused after damage. However, it is still necessary to find specific and more effective alternatives aimed at recovering lost cardiac functionality. Experimental evidence suggests that LX treatment may ameliorate myocardial injury outcome. Chen et al. were the first to describe a protective role for LXA_4_ in a rabbit model of myocardial I/R following cardiac arrest. I.v. administration of this SPM inhibited the expression of pro-inflammatory cytokines, reducing the apoptosis of cardiac cells (77). In addition, Zhao et al. demonstrated that LXA_4_ preconditioning and post-conditioning (i.v.) in myocardial I/R injury attenuated myocardial metabolic disturbance, inhibiting the inflammatory reaction and oxidative stress (78). This protective mechanism appears to occur by a downregulation of caspase 12 and GRP-78, both implicated in apoptosis (79).

### Myocarditis

Myocarditis is a pathology caused by the inflammation of the cardiac muscle that can be associated to viral infections, toxic substances, or autoimmune processes. It is also considered one of the main precursors of dilated cardiomyopathy and one of the main causes of cardiac transplant in young adults (80).

LXs exhibited a protective effect in murine models of myocarditis. Part of this effect is achieved by cardiac inhibition of PI3K/Akt and NF-κB pro-inflammatory pathways (81), whose deleterious roles in cardiac pathology have been widely described (82, 83). Furthermore, the activation of NRF2 antioxidant response represents one of the main effects of LX-mediated protection as observed in a myocarditis model. We recently described that in cardiomyocytes, this activation occurs *via* CaMKK2-AMPKα pathway (84). Interestingly, patients with severe heart failure also exhibit decreased plasma levels of LXs, indicating deficient resolution of inflammation (n = 18 mild-to-moderate *vs*. 16 severe chronic heart failure) (85).

Altogether, current evidence demonstrates that LXs are able to efficiently coordinate the pro-resolutive response in the heart, suggesting that LX-based therapy could be a more effective alternative to treat cardiac diseases preventing cardiac inflammation, remodeling and dysfunction.

## Respiratory Diseases

### Acute Lung Injury

Acute lung injury (ALI), and its most severe form, acute respiratory distress syndrome (ARDS) are manifestations of the lung to an inflammatory response and have high morbidity and mortality in the present (86). They are characterized by severe hypoxemia, hypercapnia, diffuse infiltration in the chest X-ray and a substantial reduction in pulmonary compliance ultimately leading to respiratory failure (86).

Inflammation and activation of immune cells are critical for ALI and ARDS development, since the release of pro-inflammatory cytokines and proteases increases alveolar-capillary barrier permeability, disrupting the appropriate clearance of alveolar fluid and leading to pulmonary edema (87, 88). In this context, research in animal models of ALI revealed that i.v. LXA_4_ treatment diminished the production of pro-inflammatory cytokines and ROS, thus improving alveolar fluid clearance (89–91). BML-111 (i.p.) and ATL (i.v.) exhibited similar results including preventing neutrophil infiltration, promoting its clearance (92, 93) and inhibiting the formation of neutrophil-platelet aggregates (87).

LXs and analogs also modulate pulmonary cells by preventing apoptosis and promoting proliferation of alveolar type II cells (94), reducing inflammatory signaling in microvascular endothelial cells (95, 96) and promoting autophagy in alveolar macrophages (97). In addition, they diminish fibrosis and support appropriate alveolar epithelial repair by inhibiting collagen production and proliferation of lung fibroblasts (94). LXs regulate these processes by antagonizing TLR4/NF-κB and MAPK/AP-1 signaling (95, 96, 98) and by activating both the anti-inflammatory and anti-fibrotic ACE2-Ang- (1–7)-Mas axis, mainly through an upregulation of levels and activity of ACE2 (90, 99) and the antioxidant NRF2/HO-1 pathway (96, 100). Altogether, the ability of LXs to maintain the integrity of pulmonary epithelia and to prevent infiltration of immune cells accentuates their therapeutic potential in relation to ALI and ARDS, especially by blunting neutrophilia, the hallmark of these pathologies.

### Asthma

Asthma is characterized by acute episodes of shortness of breath, coughing and chest tightness due to an underlying chronic inflammatory process sustained by a combined action of immune cells and bronchial epithelial cells (101). It affects 300 million people worldwide, being one of the most common chronic diseases in children and adults (102). Corticosteroid treatment is the most common medication for asthma, however, a small percentage of patients develop a more severe form that is unresponsive to this treatment, demanding more effective alternative therapies (103).

The potential of LXs to serve not only as a treatment but also as a biomarker for asthma has been extensively examined, as LXs concentration can be easily evaluated in sputum or exhaled breath. Decreased LXA_4_ levels have been correlated with increased asthma severity in both adults (n = 12 mild asthma, 15 moderate, 24 severe and 10 healthy) (40, 104) and children (n=36 mild asthma, 42 moderate, 28 severe *vs*. 40 healthy) (105–107). In fact, alveolar macrophages obtained from patients with severe asthma synthesize less LXs *per se* and in response to LPS treatment (n=11 severe asthma, 12 non-severe and 14 healthy) (103). In severe asthmatic children, alveolar macrophages were also found to be apoptotic and less functional (n=28 severe *vs*. 10 healthy) (108). This impaired LX synthesis explains in part the neutrophilia and eosinophilia observed in asthma, especially in severe cases (103). Indeed, LXs and analogs have been observed to attenuate eosinophil function as well as T lymphocyte and mast cell activity, both *in vitro* and *in vivo* (109–111). In relation to this, LXs managed to enhance NK cells functions in patients with asthma (112), including NK-induced apoptosis in eosinophils preventing enhanced inflammation (113).

Notably, 5-10% of asthmatic patients suffer from aspirin-intolerant asthma (AIA), developing exacerbated inflammation and asthmatic attacks upon aspirin treatment in contrast to aspirin-tolerant asthmatics (ATA) (114). LXs may play a major role in this syndrome since Sanak et al. proved that aspirin-treated blood from AIA patients produced less LXA_4_ and ATL than blood from ATA patients (n= 14 AIA *vs*. 11 ATA) (114). Similarly, urinary ATL levels were lower in AIA patients when compared to ATA patients (n=15 AIA vs. 16 ATA) (115). An interesting approach would be evaluating whether exogenous LX administration could ameliorate symptoms in AIA patients by balancing LX deficiency.

In light of the encouraging results obtained, recent advances in asthma treatment include a pilot application based on the inhalation of two LX analogs to treat children with acute episodes of asthma, which turned out to be a safer and more efficient alternative than some of the current asthma medications (116).

### Idiopathic Pulmonary Fibrosis

Idiopathic pulmonary fibrosis (IPF) is a type of interstitial lung disease characterized by a progressive fibrotic process in the lungs that severely obstructs gas exchange, eventually causing respiratory failure and death, with mean survival ranging between 3-5 years (117, 118). The only treatment available is lung transplantation, with alternative therapies focusing on delaying fibrosis development and improving patients’ quality of life.

LXs have been reported to exert anti-fibrotic actions in the lung, mainly by inhibiting TGF-β signaling and collagen I production in fibroblasts (94, 119, 120), and by preventing fibroblasts proliferation and differentiation into myofibroblasts (94). These effects have also been observed in human lung myofibroblasts obtained from IPF patients, together with a reduction of α-smooth muscle actin (SMA) expression and actin stress fibers formation and contraction (121). Likewise, mice subjected to bleomycin-induced lung fibrosis and treated i.t. with ATL exhibited reduced inflammation and fibrosis, resulting in amelioration of pulmonary performance and mouse survival (122, 123).

### Cystic Fibrosis

Cystic fibrosis (CF) is an autosomal recessive disorder caused by a mutation in the gene encoding for cystic fibrosis transmembrane conductance regulator (CFTR) (124). It is one of the most common genetic diseases, with a prevalence of 1:2500 in Caucasians and a median life expectancy under 50 years of age (124, 125). Defects on CFTR lead to mucus accumulation, bacterial infection and exacerbated inflammation, eventually causing respiratory failure (124). In this sense, LXA_4_ treatment in both *in vitro* and *in vivo* (i.v.) CF models was found to reduce bacterial and neutrophil counts (126), as well as prevent epithelial barrier disruption upon pathogen infection (127).

Interestingly, CFTR and LXs appear to be profoundly interrelated. Mattoscio et al. found that inhibition of CFTR in platelets decreased LXA_4_ synthesis (128). In fact, platelets from CF patients produce 40% less LXA_4_ than healthy controls (n = 6 homozygous, n = 8 heterozygous and n = 4 healthy). CFTR-mutated animals also exhibit reduced LXA_4_ levels, whereas restoring CFTR activity augmented them (129).

LXs were also found to affect the function of other pulmonary ion transport channels inducing Cl^-^ secretion through calcium-dependent chloride channels, inhibiting Na^+^ absorption by epithelial sodium channels and activating K_ATP_ channel (130–132). As a result, LXs promote mucus clearance and airway epithelial repair, preserving airway surface liquid layer and protecting against bacterial infection and lung inflammation, which underscores their therapeutic potential to ameliorate respiratory diseases.

## Renal Diseases

### Renal Ischemia/Reperfusion Injury

Renal ischemia/reperfusion injury is caused by a sudden temporary impairment of kidney´s blood flow. It is usually associated with a robust inflammatory and oxidative stress response to hypoxia and reperfusion which impairs organ function (133). Although its pathophysiology is not completely understood, oxygen radicals generated at reperfusion phase initiates a cascade of deleterious cellular responses leading to inflammation, cell death, and acute kidney failure.

Treatment with ATLa (i.v.) was found to restore renal function and morphology and to diminish pro-inflammatory cytokine production and neutrophil infiltration in a mouse model of acute renal failure (134). Transcriptomic analysis performed in this model revealed that pre-treatment with this analog also managed to downregulate pro-fibrotic and pro-apoptotic genes, including collagen, transgelin and Fos-like proteins, while upregulating genes implicated in the antioxidant defense, cell growth and transport proteins, like glutathione, angiogenin and aquaporin (135). At the molecular level it has been proposed that this protective effect is mediated by MAPKs, mainly p38 and ERK, and PPAR/NRF2 pathways (136, 137).

### Renal Fibrosis

Renal fibrosis is mainly mediated by excessive proliferation of mesangial cells, which plays an important role in glomerular inflammation. Both i.v. administration of LXA_4_ and benzo-LXA_4_ improved renal fibrosis in rats by diminishing renal apoptosis, TNFα and IFNγ expression, TGF-β and PAI-1 activation, and collagen deposition (138). Attenuation of MAPK, Akt and Smads signaling pathways are responsible for these effects. Interestingly, Brennan et al. observed that LX upregulates the expression of let-7c miRNA to promote the anti-fibrotic response, manifesting the existence of LX-activated miRNA pathways (139). Due to their ability to inhibit adverse remodeling in the kidney, LX treatment represents a great alternative to prevent the development of renal fibrosis and subsequent chronic kidney disease.

### Diabetic Kidney Disease

Diabetic kidney disease occurs in >30% of patients with type 2 diabetes mellitus and is characterized by a maladaptive response of the renal parenchyma, which is intensified by the development of a chronic inflammatory response, finally leading to renal failure. Results from a clinical trial in chronic kidney disease patients revealed that aspirin treatment increased ATL levels in diabetic patients, however the effects of this increase were not evaluated (140). Studies in diabetic kidney disease animal models concluded that i.p. injection of LXA_4_ and benzo-LXA_4_ attenuated the development of the disease, including pro-inflammatory and pro-fibrotic signaling (141). Transcriptomic profile of this model found enrichment of classical pro-inflammatory pathways (TNFα, NF-κB, TGF-β) and identified activation of early growth response-1 (EGR-1) network to be involved in diabetic pathology as well (141). LXs managed to downregulate the transcriptional network of these mediators, thus representing an effective treatment to prevent renal inflammation and fibrosis developed in diabetic pathology.

## Periodontal Diseases

Periodontal diseases (PD) represent a broad group of diseases characterized by chronic inflammation in the supporting structures of the teeth, in particular gingivae, bones and ligaments. It is typically initiated by bacterial infection, which induces an inflammatory reaction in the gingivae –termed gingivitis– that subsequently progresses into PD when untreated (142). PD can evolve into periodontitis, a much more severe form of the disease that can ultimately cause loss of teeth. The global prevalence of PD has been estimated to range between 10-15% of the population (142).

PD has been described to be initiated mainly by the inflammation of the periodontium, which consequently induces PMN recruitment since they represent the first line of defense against bacterial infection (143). An exacerbated recruitment of these immune cells to the periodontium weakens periodontal tissue leading to PD. LXs have been widely described to ameliorate PD outcome (144) showing that in animal models are able to reduce the release of pro-inflammatory mediators as well as PMN recruitment to the affected area (145). Studies in patients have proposed that reduced serum or salivary levels of LXA_4_ in patients can be useful as PD marker (n >65) (146–148).

The protective mechanism appears to lie in the inhibition of pro-inflammatory cytokines that modulate PMNs, hampering their recruitment and pro-inflammatory signaling thus preventing the onset of periodontal inflammation (149). In this sense, LXA_4_ treatment has also been found to abolish NF-κB and TNFα signaling in periodontal ligament cells, which play a pivotal role exacerbating the inflammatory response (150). Furthermore, increased circulating ROS levels and blood aggregation caused by *P. gingivalis* infection were successfully reduced upon LXA_4_ treatment, and this effect appears to be dependent on platelet-PMN interaction (151). In a different context, this SPM has also been shown to stimulate proliferation and migration of stem cells of the apical papilla -the main source of dentin-like structures- as well as inhibiting their pro-inflammatory activation, which may indicate LXs have also regenerative potential besides their anti-inflammatory properties (152). In fact, currently, targeting PD with LX-based treatment has reached clinical trial in the form of oral rinse (https://clinicaltrials.gov/ct2/show/NCT02342691).

## Arthritis and Rheumatic Diseases

Rheumatoid arthritis (RA), an autoimmune disease causing severe destructive inflammation of the joints with associated systemic complications, is one of the most prevalent rheumatic disease to date (153). Current treatments are based on corticosteroids, potent anti-inflammatory drugs that are also well known to have adverse effects (154).

Studies in animal models of RA have reported that administration of either LXA_4_ (i.art.) or BML-111 (i.p.) attenuates arthritis in mice by diminishing joint erosion, pro-inflammatory cytokines release and immune cell infiltration (155, 156). In the same line, it was demonstrated that 12-LO/15-LO deficiency exacerbates the development of arthritis in mice partly due to a reduction in LXA_4_ levels (157). In this context, LXA_4_ treatment has been shown to prevent pro-inflammatory activation of fibroblast-like synoviocytes, the main cell type responsible for immune activation and inflammation in RA (158). Regarding the molecular pathways underlying this beneficial effect, it is known that LXA_4_ abrogates IL-6 expression (158), counteracts MMP/TIMPs imbalance in tissue degradation and fibrosis (159), and opposes IL-1β and TGF-β pro-inflammatory and pro-fibrotic actions in human fibroblast-like synoviocytes (158, 159). These effects occur in part by inhibition of p38 MAPK signaling pathway (PMID 33221976).

Besides RA, LXA_4_ treatment (i.p.) has been found to alleviate osteoarthritis in rodent models (160, 161) and its deficiency appears to be partly responsible for the onset of systemic lupus erythematosus (162). Indeed, it has been described that corticosteroids can inhibit LX production in the long term, which could explain the negative effects of the prolonged use of these drugs (163). Altogether, this evidence exhibits the crucial role that LXs and other SPMs play in rheumatic disorders as modulators of immunosuppressive and anti-inflammatory processes. Nonetheless, additional research should be conducted to strongly determine the specific effects of SPMs in rheumatic pathologies to appropriately evaluate their benefits.

## Concluding Remarks

**Figure 3:**
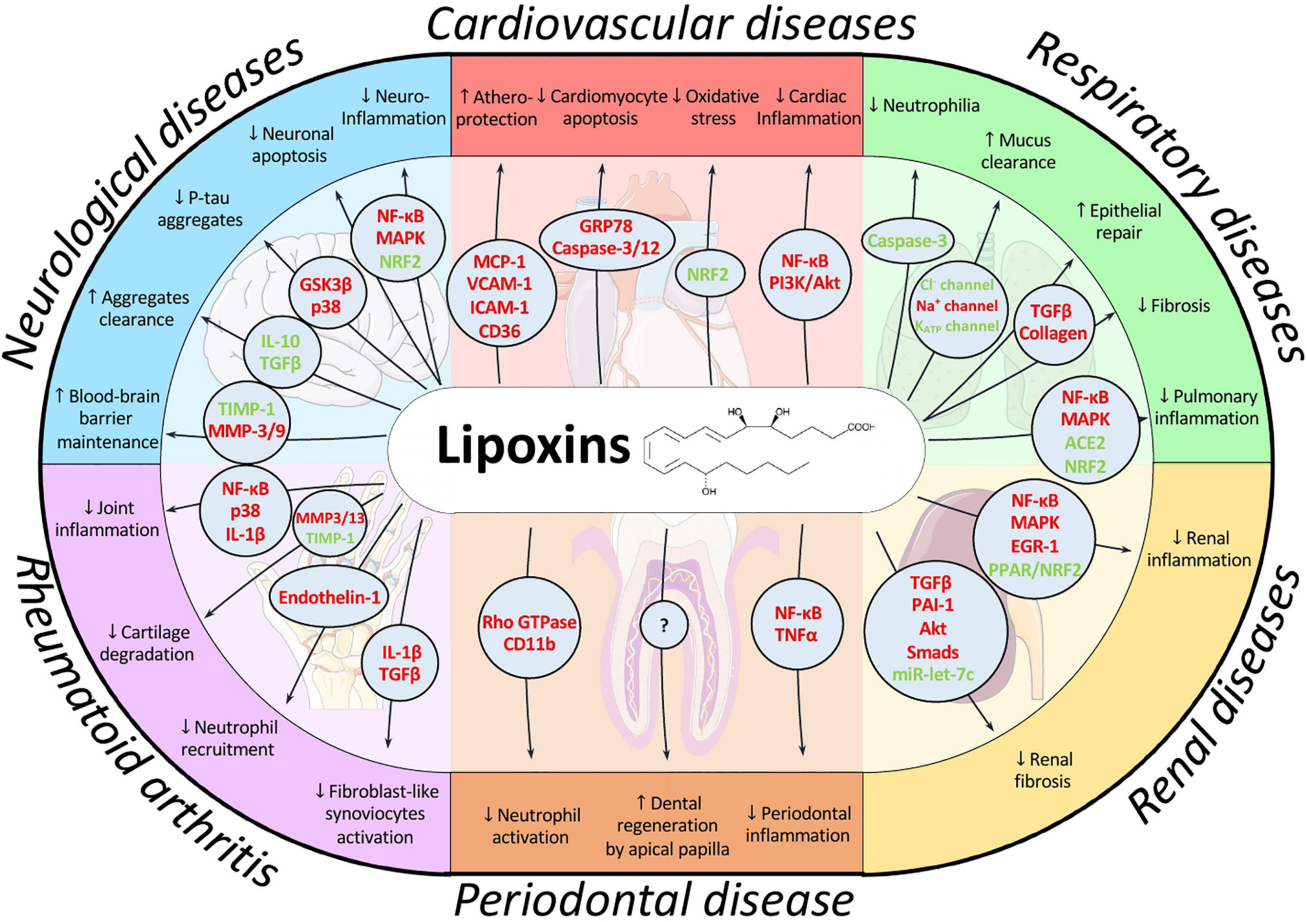
Schematic overview of the main signaling pathways modulated by lipoxins and their effects in the pathologies addressed in this review. LXs have been described to coordinate a wide range of signaling pathways depending on cell context, allowing for a fine-tune regulation of the resolution process. For example, they induce apoptosis in neutrophils to promote their clearance while they prevent apoptosis in cardiomyocytes thus exhibiting cardioprotective actions. By modulating different pathways, LXs can exert protective effects on different cell types and environments, as observed by their effects on the brain, heart, lung, liver, periodontium and joints. Mediators highlighted in red indicate downregulation or inhibition by LX action whereas green indicates upregulation or activation.

Persistent inflammation underpins many of the most prevalent pathologies at the present. Thus, a chronic inflammatory scenario is present in many cardiovascular, pulmonary, or neurological diseases as well as metabolic disorders and even cancer. LXs are endogenous pro-resolving lipid mediators whose levels are significantly reduced in a wide range of pathologies affecting the main systems we have reviewed. These mediators can play a decisive role in many of these pathologies, mainly due to their capacity to both halt the inflammatory signaling and reduce oxidative stress (**Figure 3**). Exogenous administration of LXs or stimulation of their endogenous synthesis represent interesting alternative therapies that have provided successful results in animal models. LXs exert beneficial effects at different routes of administration, and synthetic analogs with more potent and prolonged actions are commercially available. Moreover, these SPMs have more reduced side-effects than most current treatments due to their endogenous nature. All these factors make them attractive targets from a pharmacological point of view. Nonetheless, additional evaluation of pharmacokinetics as well as potential side-effects is necessary to fully determine their safety and effective dose. Altogether, LX-based therapies emerge as a promising approach to kick off resolution pharmacology, which has exhibited great potential and could provide alternatives to treat current health problems with high incidence worldwide in the near future.

## Author Contributions

RJ, SS-G, and PP wrote the manuscript. MF-V and LB provided funding and corrected the manuscript. All authors contributed to the article and approved the submitted version.

## Funding

This work was supported by Ministerio de Economía, Industria y Competitividad, Ministerio de Ciencia, Investigación y Universidades, and Agencia Estatal de Investigación (SAF2017-82436R), Centro de Investigación Biomédica en Red en Enfermedades Cardiovasculares (CB16/11/00222), Consorcio de Investigación en Red de la Comunidad de Madrid, S2017/BMD-3686 and Fondo Europeo de Desarrollo Regional. RI holds a FPU PhD fellowship of the Ministerio de Ciencia, Investigación y Universidades (FPU16/00827).

## Conflict of Interest

The authors declare that the research was conducted in the absence of any commercial or financial relationships that could be construed as a potential conflict of interest.

## Abbreviations

ACE2, Angiotensin-converting enzyme 2; AD, Alzheimer disease; AIA, Aspirin-intolerant asthma; ALI, Acute lung injury; ALXR, Lipoxin receptor; AMPK, AMP-activated protein kinase; AP-1, Activator protein 1; ARDS, Acute respiratory distress syndrome; ATA, Aspirin-tolerant asthma; ATL, Aspirin triggered lipoxin; ATLa, 15-epi-16-(p-fluoro)-phenoxy-​lipoxin A_4_; CaMKK2, Calcium/calmodulin dependent protein kinase kinase 2; CF, Cystic fibrosis; CFTR, Cystic fibrosis transmembrane conductance regulator; ERK, Extracellular signal-regulated kinases; GSK3β, Glycogen synthase kinase 3 beta; HO-1, Heme-oxygenase 1; i.art., intra-articular; ICV, Intracerebroventricular; IFNγ, Interferon gamma; IL, Interleukin; i.p., Intraperitoneal; IPF, Idiopathic pulmonary fibrosis; I/R, Ischemia/Reperfusion; i.t., Intratracheal; i.v., Intravenous; JNK, c-Jun N-terminal kinase; LO, Lipoxygenase; LX(s), Lipoxin(s); LXA_4_, Lipoxin A_4_; LXA_4_-ME, LXA_4_-methyl ester; MAPK, Mitogen-activated protein kinase; MMP, Matrix metalloproteinase; NF-κB, Nuclear factor κB; NK, Natural killer cells; NRF2, Nuclear factor erythroid 2-related factor 2; PD, Periodontal disease; PI3K, Phosphatidylinositol 3-kinase; PMN, Polymorphonuclear cell; PPAR, Peroxisome proliferator- activated receptors; s.c., Subcutaneous; SPM, Specialized pro-resolving mediators; TGF-β, Transforming growth factor beta; TIMP, Tissue inhibitor of metalloproteinase; TNFα, Tumor necrosis factor alpha; RA, Rheumatoid arthritis; ROS, Reactive oxygen species.
